# HIV Protease Hinge Region Insertions at Codon 38 Affect Enzyme Kinetics, Conformational Stability and Dynamics

**DOI:** 10.1007/s10930-023-10132-6

**Published:** 2023-07-08

**Authors:** Zaahida Sheik Ismail, Roland Worth, Salerwe Mosebi, Yasien Sayed

**Affiliations:** 1 Protein Structure-Function Research Unit, School of Molecular and Cell Biology, University of Witwatersrand, Johannesburg, 2050 South Africa; 2 College of Agriculture & Environmental Sciences, School of Agriculture and Life Sciences, Department of Life and Consumer Sciences, UNISA, Pretoria, South Africa

**Keywords:** HIV-1, Protease, enzyme kinetics, molecular dynamics, conformational stability

## Abstract

HIV-1 protease is essential for the production of mature, infectious virions and is a major target in antiretroviral therapy. We successfully purified a HIV-1 subtype C variant, L38↑N↑L^− 4^, containing an insertion of asparagine and leucine at position 38 without the four background mutations - K20R, E35D, R57K, V82I using a modified purification protocol. Isothermal titration calorimetry indicated that 50% of the variant protease sample was in the active conformation compared to 62% of the wild type protease. The secondary structure composition of the variant protease was unaffected by the double insertion. The specific activity and *k*_cat_ values of the variant protease were approximately 50% lower than the wild type protease values. The variant protease also exhibited a 1.6-fold increase in *k*_cat_/*K*_M_ when compared to the wild type protease. Differential scanning calorimetry showed a 5 °C increase in T_m_ of the variant protease, indicating the variant was more stable than the wild type. Molecular dynamics simulations indicated the variant was more stable and compact than the wild type protease. A 3–4% increase in the flexibility of the hinge regions of the variant protease was observed. In addition, increased flexibility of the flaps, cantilever and fulcrum regions of the variant protease B chain was observed. The variant protease sampled only the closed flap conformation indicating a potential mechanism for drug resistance. The present study highlights the direct impact of a double amino acid insertion in hinge region on enzyme kinetics, conformational stability and dynamics of an HIV-1 subtype C variant protease.

**Supplementary Information:**

The online version contains supplementary material available at 10.1007/s10930-023-10132-6.

## Introduction

Acquired immunodeficiency syndrome (AIDS) is caused by the pathogenic Human Immunodeficiency Virus (HIV) and continues to remain a major global health concern. In 2020, 1.5 million new infections were reported with a total of 37.7 million people infected worldwide [1]. HIV is of particular concern in eastern and southern Africa because these regions contributed approximately 55% to the global number of infections in 2020 [1]. Disturbingly, South Africa has 7.8 million people infected with HIV, making this region the epicentre of the HIV/AIDS pandemic.

The HIV replication cycle is a complex multi-step process requiring various proteins. The HIV-1 protease is crucial for the production of new infectious virus particles and, therefore, provides an appealing target for drug therapy. HIV-1 protease is released from the Gag/Gag-Pol precursor through autoproteolytic processing. The protease is a 22 kDa homodimer consisting of two monomers of 99 amino acids each [2]. The two monomers associate by means of non-covalent interactions contributed by amino acid side chain residues positioned at the dimer interface [3]. The HIV-1 protease can only function in its dimeric form with the Asp25 from each monomer constituting the main catalytic residue forming the active site cavity at the dimer interface [4, 5].

The South African HIV-1 subtype C (C-SA) protease differs from the subtype B consensus sequence by eight naturally occurring polymorphisms (NOPs)– T12S, I15V, L19I, M36I, R41K, H69K, L89M and I93L [6]. These NOPs occur distal from the active site and does not affect the catalytic activity, substrate binding affinity, viral fitness or structural stability [7]. However, these NOPs have an effect on the binding thermodynamics of available protease inhibitors (PIs) and may exacerbate drug resistance attributed to other known mutations.

**Fig. 1:**
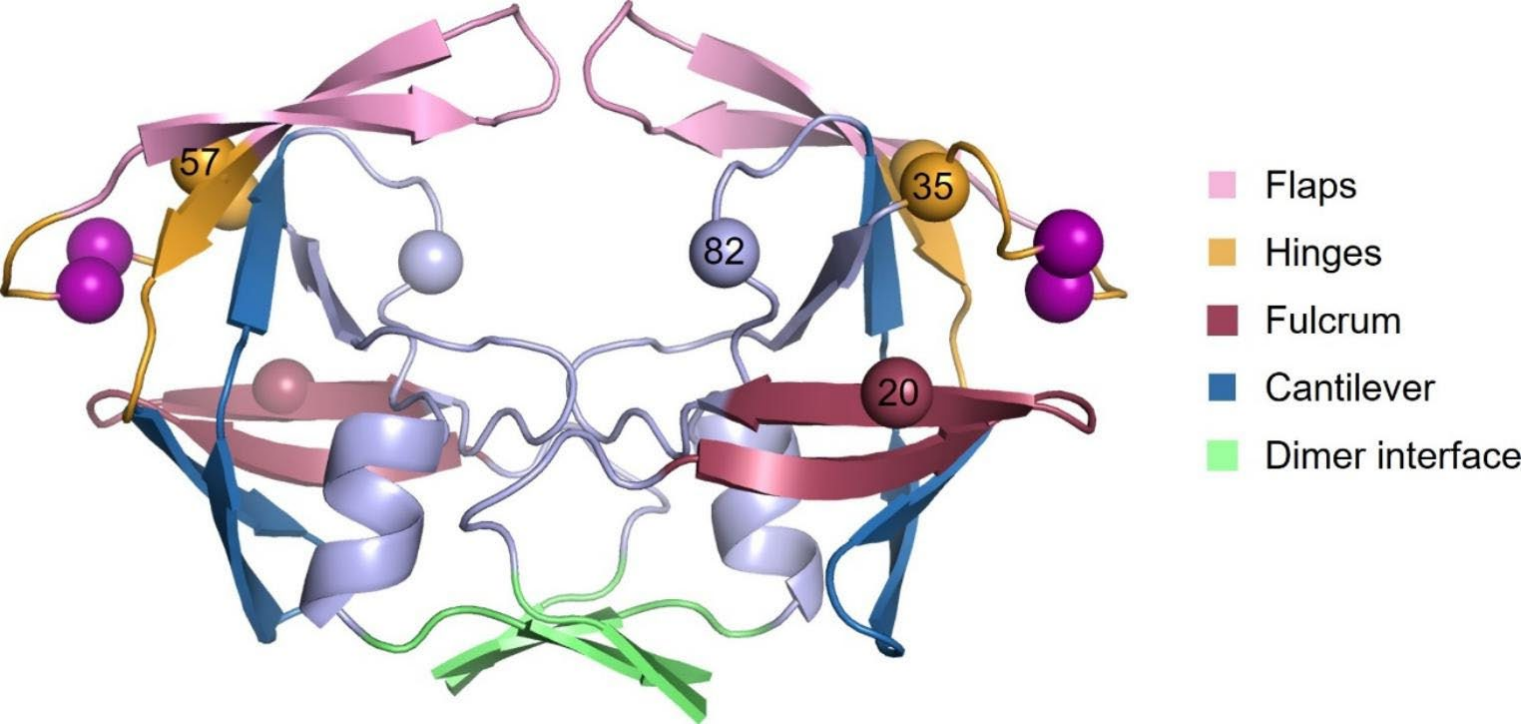
Homology model of the L38↑N↑L protease. The hinge region double insertion of Asn and Leu is shown as purple spheres, with the accompanying subset of background mutations (K20R, E35D, R57K, V82I). The image was constructed using the PyMOL v0.99rc6 software using PDB ID: 3U71 as a template

Structurally, HIV-1 protease is divided into the flap, hinge, cantilever, fulcrum, and dimer interface regions (Fig. 1). The dimer interface contains four short anti-parallel β-strands and is formed by residues 1–4 and 96–99 from the N- and C- termini from each monomer. The flap region (residues 42–56) is important for the specificity and activity of the HIV-1 protease [5] given that these glycine rich regions are flexible and control access of the polyprotein substrate or inhibitors to the active site. These flexible flaps exist in the open, semi-open and closed conformations which are defined by the distance between the flap tip Ile50/50′ residues and the catalytic Asp25/25′ residues [8]. A distance < 17 Å between these two residues represents the closed conformation, 17–22 Å represents the semi-open conformation and > 22 Å represents the open conformation [9]. The movement of the flaps is critical in regulating the open and closed flap conformations which allows the entry of substrates to the active site and subsequent binding, respectively [10]. The movement of the flaps is also largely facilitated by the hinge (residues 35–42 and 57–61), cantilever (residues 62–78) and fulcrum (residue 10–22) region [5, 11, 12]. The interconnected movement of these regions is referred to as the hydrophobic sliding mechanism and is described as the downward sliding of the hinge and cantilever regions across the surface of the fulcrum region. Consequently, the flap dynamics relies on the hydrophobic sliding mechanism and the hydrophobic core of the protease which is stabilised by van der Waals contacts that can easily be exchanged between adjacent residues [9, 13].

The hydrophobic core of the protease has limited accessibility to solvent and is composed of twenty residues, many of which are involved in the hydrophobic sliding mechanism [12, 14]. For instance, in the closed formation, the hinge region residues M36 and L38 and the cantilever residues I62, I64 and V75 form contacts with the fulcrum residue I15 [9]. Sliding of the hinge and cantilever regions across I15 and downwards toward the termini results in the transfer of the original contacts to I13 resulting in the opening of the flaps [12]. An increase in flap region flexibility could possibly reduce drug susceptibility and the rate of substrate proteolysis [5]. Additionally, mutations of the hinge residues M36 and L38 could hinder the hydrophobic sliding mechanism resulting in a shift of flap conformations. Therefore, it is imperative to understand the role of amino acid substitutions, mutations and insertions in the flap and hinge regions and the resulting contribution to the activity of the HIV-1 protease.

Though still rare (approximately 0.5% prevalence), amino acid insertions in the South African subtype C protease hinge region are being discovered more often and are frequently found in treatment-naïve patients [15]. Amino acid insertions near residues 18, 25, 36, 70 and 95 in the HIV-1 protease have been identified and appear to be duplicates of the adjacent genetic sequence [16–18]. These HIV-1 protease insertions are caused by the stalling and slippage of reverse transcriptase [19] and can also be selected for during treatment with antiretrovirals [20]. The role of these insertions has been suggested to contribute to PI resistance in the presence of additional mutations usually found in the Gag or Gag-Pol proteins [16, 21, 22]. When found in combination with other protease mutations the presence of amino acid insertions has been shown to decrease PI susceptibility by affecting the flaps and binding pocket [23]. Notably, these insertions also moderately enhance viral replication [16, 23]. Insertions between residues 35–42 in the hinge region have become increasingly more prevalent [24] considering that they are linked to impaired flap dynamics during PI binding and contribute to a decrease in drug susceptibility [5, 23, 25, 26].

A South African subtype C protease, designated L38↑N↑L, carrying several polymorphisms was isolated from a drug naïve-infant whose mother was exposed to reverse transcriptase inhibitors (Department of Health Science, Africa) [15]. The polymorphisms include a subset of mutations - K20R, E35D, R57K, V82I and a double insertion of asparagine and leucine in the hinge region at position 38 [27] resulting in each protease monomer consisting of 101 amino acids (Fig. 1). While amino acid insertions in conjunction with other mutations has been shown to affect PI therapy, very little is known about how these insertions directly affect the protease. The aim of this study was to determine how the L38↑N↑L hinge region insertions affects both the structure and function of the HIV-1 subtype C protease. Therefore, based on prior knowledge relating to the stability variant [28], which exhibited a 50% and 42% reduction in *k*_cat_ and specific activity, respectively, we studied the effect of the hinge region insertions on the HIV-1 subtype C protease. A L38↑N↑L variant containing only the double hinge region insertions and no background mutations (L38↑N↑L^− 4^) was used in this study.

## Materials and Methods

### Overexpression of Proteases

The subtype C and L38↑N↑L protease sequence data were obtained from Professor Lynn Morris (AIDS Virus Research Unit, NICD of Johannesburg, South Africa). The sequence encoding the L38↑N↑L^− 4^ variant was inserted into a pET-11b plasmid (GenScript, USA). For simplicity, the subtype C protease and L38↑N↑L^− 4^ variant protease will henceforth be referred to as the WT and variant protease, respectively. Both the WT and variant plasmids were generated with the Q7K point mutation to decrease autoproteolysis [46].

*Escherichia coli* BL21 (DE3) pLysS cells were transformed with either the pET-11a or pET-11b plasmid and were induced to express the proteases as inclusion bodies by the addition of isopropyl β-D-1- thiogalactopyranoside (IPTG) as previously described [5]. Following overexpression of the protease, cells were disrupted by sonication and centrifuged. The pellets containing the proteases were washed with 10 mM Tris, 2 mM EDTA and 2% Triton X-100, 1 mM PMSF (pH 8.0). The protease was recovered from the inclusion bodies using unfolding buffer (8 M urea, 10 mM Tris-HCl and 2 mM dithiothreitol (DTT) (pH 8.0)).

### Purification of Proteases

The WT supernatant containing the unfolded protease was refolded by dialysis against refolding buffer (10 mM formic acid, 0.01% (w/v) sodium azide and 10% glycerol (v/v)) at 4 °C for 4 h with gentle spinning. The protease was then dialysed against equilibration buffer (10 mM sodium acetate, 0.01% (w/v) sodium azide, 2 mM DTT (pH 5.0)) at 4 °C for 16 h. The WT protease was purified using CM-Sepharose ion-exchange column chromatography with a 0–1 M NaCl gradient elution. Lastly, the protease was dialysed against storage buffer (10 mM sodium acetate, 0.01% (w/v) sodium azide (pH 5.0)) at 4 °C for 16 h. Purity of the WT protease was assessed by tricine SDS-PAGE [47].

The purification of the variant protease was performed using a modified protocol published by Sherry et al. (2020) which uses a two-step purification method as shown in Figure S3. Briefly, a DEAE-column was connected in tandem to a CM-Sepharose column and equilibrated using unfolding buffer. The supernatant containing the protease was passed through the columns (DEAE and the CM-Sepharose), followed by re-equilibration using the unfolding buffer. The DEAE-column was removed and all bound proteins, including the protease, was eluted from the CM-Sepharose column in a single step using 1 M NaCl prepared in unfolding buffer. The single collected fraction was diluted 1:10 with unfolding buffer and dialysed against refolding buffer at 4 °C for 4 h with gentle stirring. The protease was then dialysed against equilibration buffer at 4 °C for 16 h. The dialysate was centrifuged at 23 000 xg for 30 min, 4 °C to remove any aggregates. The variant protease was then purified using CM-Sepharose ion-exchange column chromatography with a 0–1 M NaCl gradient elution prepared in equilibration buffer. Lastly, the protease was dialysed against storage buffer at 4 °C for 16 h. Purity of the variant protease was assessed by tricine (16%) SDS-PAGE [47]. The concentration of both proteases was determined from absorbance spectra obtained on a Jasco V-630 Spectrophotometer using a molar extinction coefficient of 24 980 M^− 1^ cm^− 1^ [48]).

### Active Site Determination of Proteases

The percentage of available active enzyme was investigated using isothermal titration calorimetry (ITC) as previously described with minor modifications [4]. Briefly, 100 µM of acetyl pepstatin was titrated (5 µl injections) into a solution of 20–25 µM protease with a stir rate of 100 rpm, an initial and final baseline of 200 and 300 s between each injection. ITC experiments were performed at 20 °C using a Nano ITC low volume instrument (TA® Instruments, New Castle, USA). A control titration (ligand into buffer) was performed to monitor the heats of dilution (HOD). The HOD were subtracted from the resulting data set, the baseline was corrected and the change in heats were fitted to an independent binding model using the NanoAnalyze™ software. The percentage of active protease in the WT and variant proteases samples was determined from the n-value with a value of 1 representing 100% active enzyme in protein preparations.

### Secondary Structure Analysis

The secondary structure of the variant protease was investigated using far-UV circular dichroism (CD). Ellipticity measurements were obtained at wavelengths ranging from 200 to 250 nm using a 1 nm bandwidth and data pitch of 0.2 nm on a Jasco J-1500 spectropolarimeter at 20 °C. The resulting spectra were averaged over 10 accumulative scans with contributions by the buffer subtracted from the collected data and subsequently converted to mean residue ellipticity (MRE).

### Steady-State Enzyme Kinetics

The kinetic parameters catalytic efficiency (*k*_cat_/*K*_M_), turnover number (*k*_cat_) and specific activity were determined in separate experiments as previously described [28]. The hydrolysis of a fluorogenic substrate (Abz-Arg-Val-Nle-Phe(NO_2_)-Glu-Ala-Nle-NH_2_) which mimics the conserved capsid/p2 cleavage site within the HIV-1 Gag polyprotein was observed. For all kinetic measurements, an excitation wavelength of 337 nm and an emission wavelength of 425 nm was used with 1 min measurement intervals during steady state. All assays were performed in triplicate at 20 °C using a Jasco FP-6300 spectrofluorometer.

The specific activity and *k*_cat_ were determined using a constant substrate concentration of 50 µM and a range of active enzyme amounts from 1 to 10 pmol. The catalytic efficiency (*k*_cat_/*K*_M_) was determined using a constant active enzyme concentration of 50 nM and a range of substrate concentrations from 1 to 10 µM. All activity assays were performed in 50 mM sodium acetate and 1 M NaCl (pH 5.0).

### Differential Scanning Calorimetry

The thermal stability the WT and variant proteases was measured as a function of temperature using the Nano-differential scanning calorimetry (DSC) microcalorimeter (TA Instruments, Delaware, USA). Prior to DSC experiments, the protein samples (30 µM) and reference solutions were degassed under a vacuum of 0.3–0.5 atm for 15 min to avoid bubble formation during sample loading. The proteases were heated linearly from 20 to 130 °C at a rate of 1 °C/min with the reference cell containing storage buffer. Data were analysed using the NanoAnalyze™ software package according to the best fitted model.

### Molecular Dynamics Simulations

All atom molecular dynamics (MD) simulations are the standard method used to simulate the dynamic motion of protein molecules *in silico* [49, 50]. MD simulations were performed on the WT and variant proteases using the Desmond software version 11.2 that was operated on LINUX architecture. All MD simulations were performed on two 3.68 GHz Intel core i7 5960x computers. The OPLS3 force field was used for both WT and variant models [51]. Using the TIP3 solvation model of the System Builder model, simulations were performed with explicit solvent in a cubic box universe [52]. The cubic box universe was selected as previously defined whereby the protein was positioned 10 Å away from the edges of a cubic box [5, 26]. Following solvation, the systems were relaxed through energy minimisation by bringing the temperature to equilibrium using the constant number of particles, volume and temperature (NVT) ensemble. Thereafter, the system was left to equilibrate for 100 ps as the temperature increased from 10 K and plateaued at 300 K. The pressure of the system was then brought to equilibrium by using the constant number of particles, pressure and temperature (NPT) ensemble. The system was left to equilibrate for 100 ps until the density of the system was stable over time [53, 54]. Once the system was equilibrated, MD simulations were performed over 50 ns with data collected over 100 frames, every 0.05 ns. Analysis of the resultant trajectories was performed using the Simulation Quality Analysis Module in Maestro (Maestro, Schrodinger LLC 2020, USA).

## Results

### Protease Preparation

Ion-exchange chromatography was used to obtain pure WT and variant proteases. Purification of the South African subtype C protease from inclusion bodies was previously developed in our laboratory [5]. While this WT purification method successfully produces pure protein, the resulting protein yield is relatively low particularly when used to purify variant proteases. As a result, the two-step purification method [29] was attempted to purify the variant protease. On its own this method resulted in some aggregation during dialysis, increased autoproteolysis and was unable to yield pure protein. An adapted protocol combining the WT purification method and the two-step purification method was, therefore, created to successfully purify the variant protease. To minimise aggregation during dialysis, the refolding buffer used for the variant protease purification contained the reagents used by Naicker et al., (2013) which contained 10 mM Tris (pH 7.5), 2 mM dithiothreitol (DTT), 0.02% (w/v) sodium azide and 10% (v/v) glycerol. To eliminate aggregation of the variant protease, the protein sample was dialysed against the WT refolding (10 mM formic acid, 0.01% (w/v) sodium azide and 10% glycerol (v/v)) and equilibration (10 mM sodium acetate, 0.01% (w/v) sodium azide, 2 mM DTT) buffer. Thereafter, instead of using a DEAE-column as in the two-step protocol, a CM-Sepharose column was used in the second purification step and by using a gradient elution (0–1 M NaCl) the contaminating proteins were separated from the protease. Finally, the storage buffer in the two-step method contained 150 mM NaCl. The presence of salt has been shown to increase the autoproteolytic activity of proteases [30] and was, therefore, removed from the variant protease storage buffer. Ultimately, the combination of changing the refolding buffer during the second purification step and omitting NaCl from the storage buffer resulted in pure, homologous variant protease. In this study, an expected monomeric weight of ~ 11 kDa was observed for both the WT and variant proteases and based on the densitometry of the bands the purity of the proteases was estimated to be > 99% pure (Figure S1). The successful purification of the WT and variant proteases allowed for further characterisation of the protease.

### Determination of Native Folded Protease

To account for autoproteolysis, the percentage of protease in its native folded conformation was determined by performing an active site titration using ITC and the aspartyl protease inhibitor, acetyl pepstatin. As indicated by the stoichiometry values, the percentage of the WT and variant protease in the native conformation was measured to be 62% and 50%, respectively (Figure S2).

The free energy related with the binding of acetyl pepstatin to the variant protease was 3 kJ/mol lower than the WT protease, Table 1. The contribution of Δ*H* and TΔ*S* to the reaction was significantly different between the two proteases. Specifically, the Δ*H* and Δ*S* values for the WT were 51.37 kJ/mol and 297 J/mol.K while the variant was 11.94 kJ/mol and 152.4 J/mol.K. The *K*_d_ values for the WT and variant were 0.44 and 1.5 µM, respectively, indicating moderate binding between the inhibitor and proteases. However, it should be noted that the binding of the variant protease was slightly weaker in comparison to the WT as represented by the larger *K*_d_ value.

**Table 1:** Comparison of acetyl pepstatin binding parameters between the WT and variant proteases

Acetyl pepstatin	Δ*H* (kJ/mol)	Δ*S* (J/mol.K)	Δ*G* (kJ/mol)	*K*_d_ (µM)
**WT**	51.37	297	-36	0.44
**Variant**	11.94	152.4	-33	1.5

### Secondary Structure Analysis

**Fig. 2:**
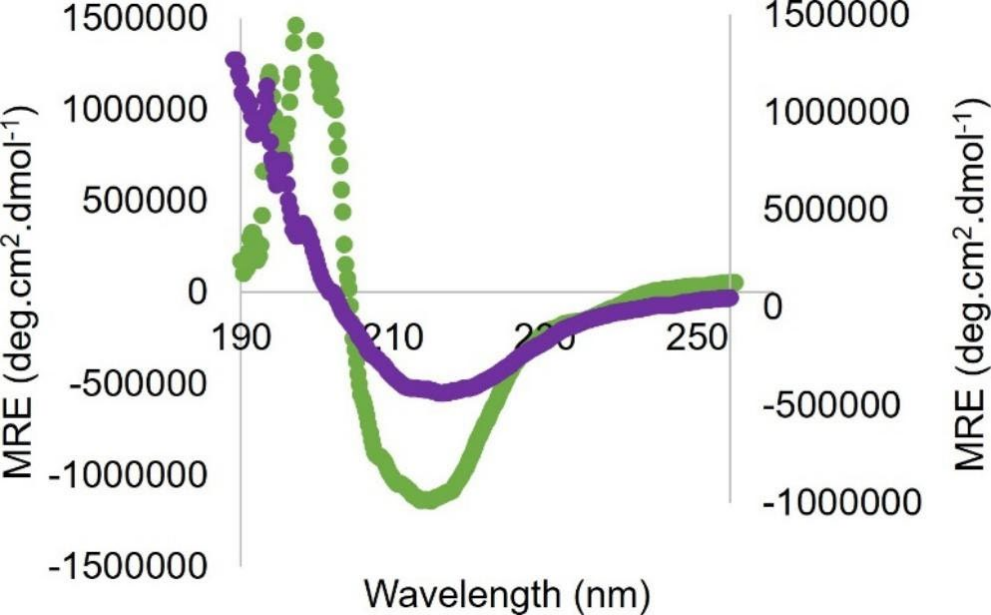
Far-UV spectrum of the proteases ranging from 190–250 nm. The green line represents the WT protease, and the purple line represents the variant protease. WT CD spectrum displaying an ellipticity minimum and maximum at 218 and 195 nm, respectively. The variant protease displays an ellipticity minimum at 218 nm. CD measurements were determined in storage buffer at 20 °C. The y-axis on the left corresponds to the WT and the y-axis on the right corresponds to the variant protease

The secondary structure of the dimeric variant protease was investigated and compared to the WT protease using far-UV CD. The far-UV CD measurements for both WT and variant exhibited a trough at 218 nm, Fig. 2, which is characteristic of a primarily β-sheeted protein. The reported measurements were normalised for number of amino acids, concentration and path length of the cuvette used for measurements. The results obtained agreed with the wild type subtype C crystal structure (PDB: 3U71) with the WT and variant proteases being a predominantly β-sheeted protein [5].

### Steady-State Enzyme Kinetics

The steady-state enzyme kinetics, Table 2, of the apo WT and variant proteases was determined using a fluorogenic substrate. The substrate mimics the natural polypeptide’s capsid/p2 cleavage site (KARVL/AEAM) allowing for cleavage of the fluorescent substrate. The specific activity and turnover number of the variant was approximately 50% lower than the WT protease. In particular, the *k*_cat_ of the variant and the WT was 5.0 and 9.1 s^− 1^, respectively, while the specific activity was 12.7 and 22.6 µmol.min^− 1^.mg^− 1^, respectively. The catalytic efficiency (*k*_cat_/*K*_M_) was 2.3 and 1.4 s^− 1^.µM^− 1^ for variant and the WT protease, respectively, indicating a 1.6-fold increase for the variant protease.

**Table 2:** Steady-state kinetics of the WT and variant proteases

	Specific activity (µmol.min^− 1^.mg^− 1^)	*k*_cat_ (s^− 1^)	*k*_cat_/*K*_M_ (s^− 1^.µM^− 1^)
**WT**	22.6 ± 2.0	9.1 ± 0.8	1.4 ± 0.2
**Variant**	12.7 ± 1.2	5.0 ± 0.6	2.3 ± 0.6

### Stability of Proteases

**Fig. 3:**
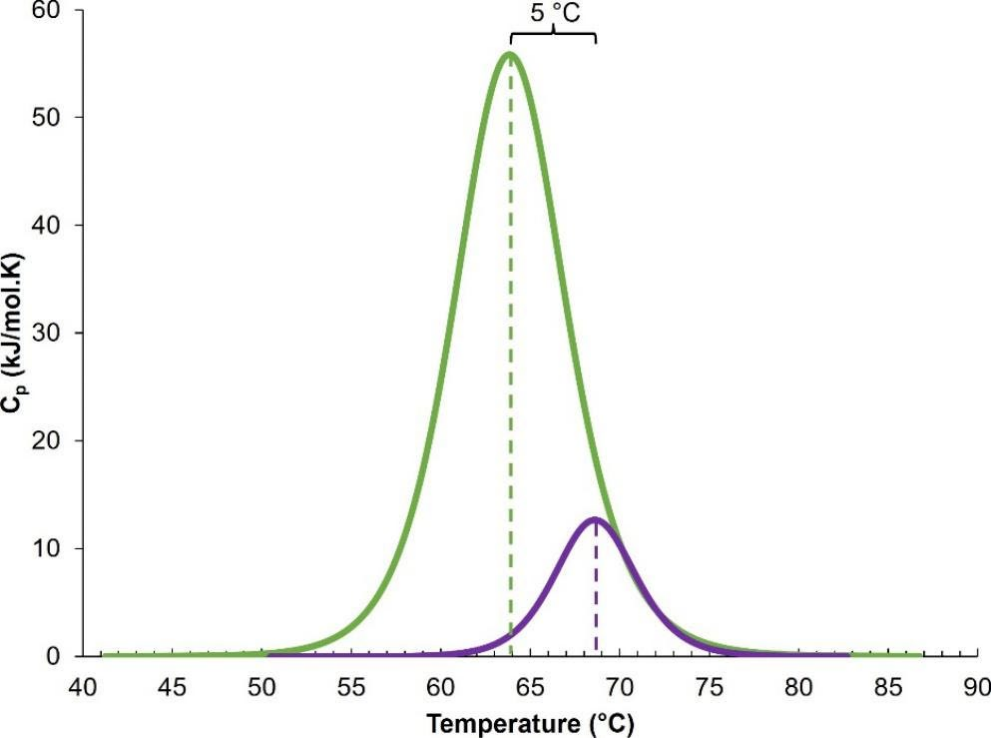
The structural stability of the WT and variant proteases. The figure shows temperature dependence of the heat capacity function for the proteases. The green line represents the WT protease, and the purple line represents the variant protease. Both experiments were performed under the same experimental conditions at a protease concentration of 30 µM in storage buffer

Differential scanning calorimetry (DSC) was used to determine the structural stability of the proteases by measuring the change in heat capacity (ΔC_p_) of the proteins in solution as a function of temperature. Noticeably, the T_m_, which corresponds to the structural stability, of the two native proteases were noticeably different, with values for the WT and variant proteases of 64 and 69 °C, respectively, suggesting that the apo variant is more stable than the WT protease, Fig. 3. Additionally, the ΔC_p_ of the variant protease (12 kJ/mol.K) was significantly lower than the WT protease (54 kJ/mol.K). The Δ*H* values associated with the WT and variant proteases were 459.3 and 643 kJ/mol, respectively.

### Molecular Dynamics Simulations

**Fig. 4:**
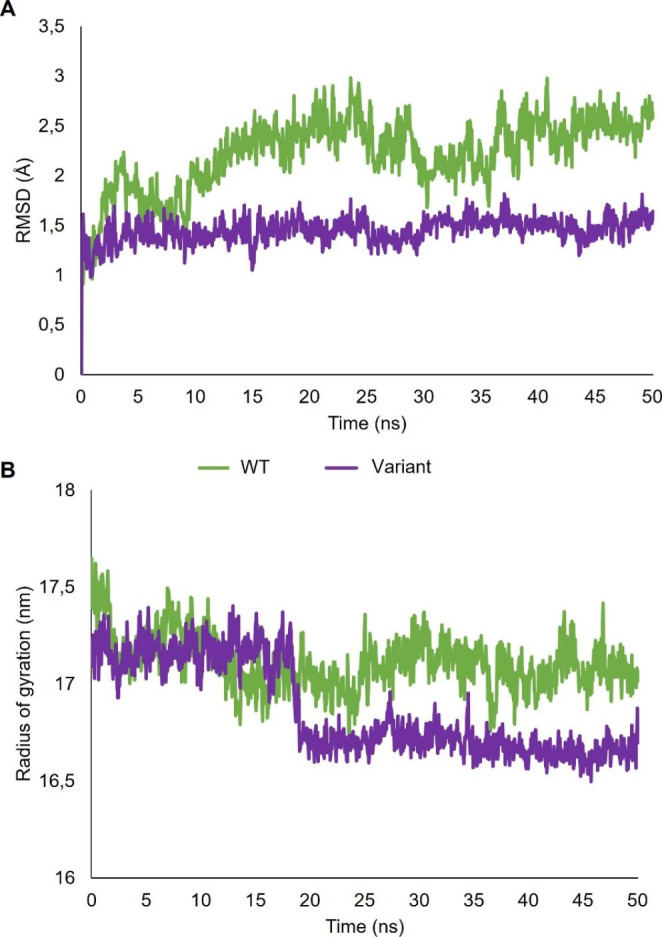
**A**: RMSD of the WT and variant protease polypeptide backbones. **B**: Radius of gyration of the WT and variant protease polypeptide backbones. The green and purple lines correspond to the WT and variant proteases, respectively

The root-mean-square deviation (RMSD) of the WT and variant was assessed to determine the overall structural stability of the proteins during the simulations (Fig. 4A). Noticeably, the RMSD data for the WT was higher than variant throughout the simulation with the variant protease fluctuating constantly around ~ 1.2 Å. The WT had an average RMSD value of 2.22 Å while the variant protease exhibited an average RMSD values of 1.45 Å indicating that this variant protease was more stable than the WT (Table 3).

The radius of gyration (R_g_) was used to assess how compact the proteases were during the simulations. Initially, the R_g_ was similar for both the WT and variant proteases at the start of the simulation (Fig. 4B). However, after 20 ns the average R_g_ indicated that the variant protease was more compact relative to the WT protease (Table 3).

**Table 3:** Average RMSD data for the WT and variant proteases over the course of 50 ns MD simulations

Protein	Average RMSD	Average R_g_
**WT**	2.22 ± 0.37	17.11 ± 0.14
**Variant**	1.45 ± 0.13	16.87 ± 0.24

### RMSF

**Fig. 5:**
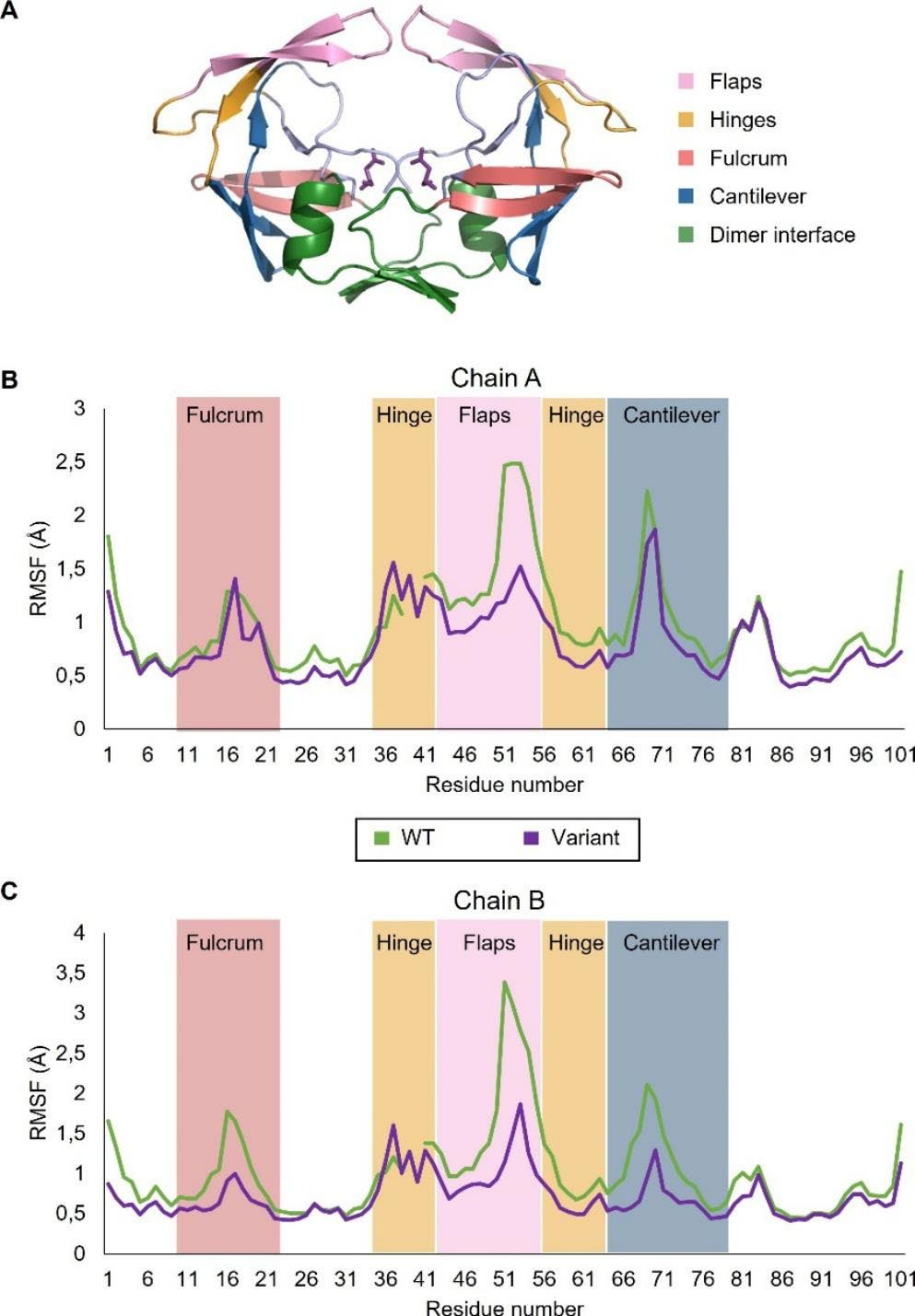
RMSF of the protease with respect to residue number. **A**: Model of protease displaying flaps (pink), hinges (orange), fulcrum (red), cantilever (blue) and dimer interface (green). **B**: RMSF of chain **(A)C**: RMSF of chain **(B)** The green and purple lines correspond to the WT and variant proteases, respectively

Root-mean-square fluctuation (RMSF) describes the average deviation of the C_α_ of each amino acid residue relative to a reference point over time. The RMSF was used to assess the local changes that occurred along both the protein chains of the WT and variant proteases (Fig. 5). The WT residues exhibiting the greatest RMSF values were 42–56 and 62–78 which corresponds to the flap and cantilever regions, respectively (Fig. 5). The most noticeable difference in the average RMSF of variant occurred in the flap region where the average RMSF of the flaps displayed a 44% decrease and 69% increase in chain A and B, respectively, when compared to the WT regions (Table 4). When compared to WT, the cantilever region of variant also showed a discrepancy between chains A and B with a 25% decrease and 60% increase, respectively. While the variant hinge region was not significantly different to the WT hinge region, it is important to note that this is the only region in which the RMSF is increased for both chains of variant.

**Table 4:** Percentage change (**increase**/**decrease**) in average RMSF data for L38 with regards to WT during a 50 ns MD simulation

	Fulcrum		Hinge		Flap		Cantilever	
	A	B	A	B	A	B	A	B
**Variant**	-17	49	3	4	-44	69	-25	60

### Flap Conformations

**Fig. 6:**
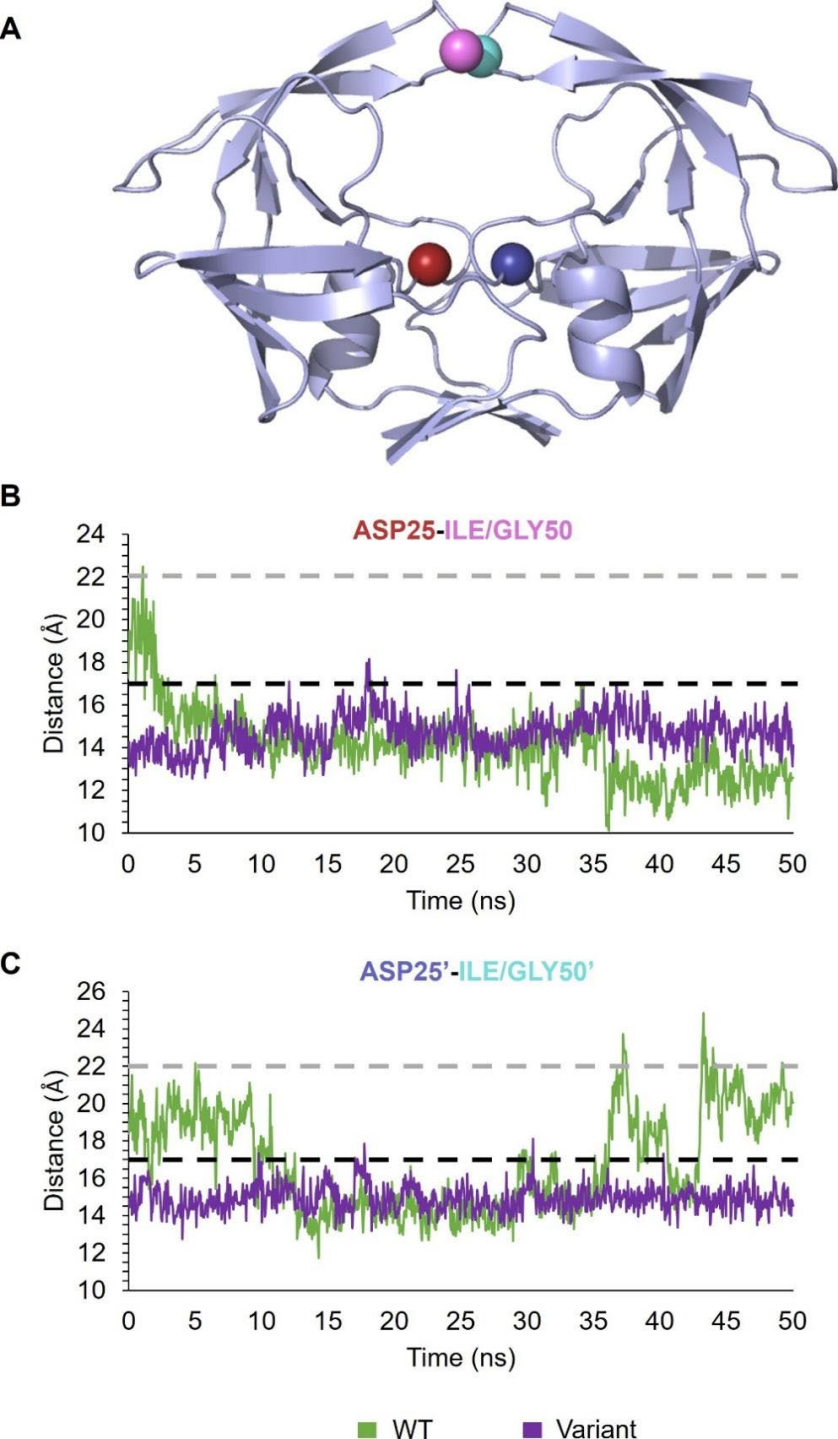
Flap conformations of the WT and variant proteases. **A**: Structure of protease displaying Asp25 (red) and Ile/Gly50 (pink) of chain A and Asp25’ (blue) and Ile/Gly50’ (cyan) of chain B. **B**: Distance between Asp25-Ile/Gly50 atoms in chain **(A)C**: Distance between Asp25’-Ile/Gly50’ atoms in chain **(B)** The black and grey dashed lines indicate 17 Å and 22 Å, respectively. The green and purple lines correspond to the WT and variant proteases, respectively

The HIV-1 protease can exist in several flap conformations namely – open, semi-open and closed which exist in an equilibrium when the protease is in the apo state [3]. These conformations can be measured using the distance between the Ile50 residue in the flap tips and the Asp25 residue found in the active site, Fig. 6A. This provides a system of measurement of the exposure of substrates or protease inhibitors to the active site cavity. As mentioned previously, these conformations are defined are < 17 Å for the closed conformation, between 17 and 22 Å for the semi-open conformation and > 22 Å for the open conformation.

The distance between Ile50 and Asp25 of each monomer was calculated for the WT (Fig. 6). Due to the insertions at position 38, all residues following this position would have shifted two places. Therefore, for the variant, the distance between Gly50 and Asp25 of each monomer was measured (Fig. 6). The distance between the flap tips and the active site for chain A of both proteases were similar at approximately 15 Å. However, the WT protease sampled the semi-open conformation at the beginning of the simulation (17–22 Å). In contrast, the distance between Ile50 and Asp25 of the WT chain B fluctuated and displayed all three conformations of the protease. The variant protease consistently measured < 17 Å for the distance calculated between Gly50 and Asp25 of chain B indicating that this protease maintained the closed flap conformation throughout the simulation.

## Discussion

The HIV-1 protease is an extremely attractive drug target considering the necessity of this enzyme to produce new infectious virions. Amino acid insertions selected by the virus during antiretroviral therapy rarely occur in the protease gene. However, more recently, insertions have become frequently observed with double insertions at the same amino acid position being observed as well [15]. Limited knowledge is currently available about the effect of these insertion polymorphisms on the overall structure, stability, dynamics and drug binding properties of the protease. Here, we report on the direct impact of the asparagine and leucine hinge region insertions on the structural stability and dynamics of the HIV-1 protease.

### Purification Using Ion-Exchange Chromatography

Combining the purification steps previously described by Naicker et al. (2013) and Sherry et al. (2020) allowed for the successful purification of this variant protease from inclusion bodies. This newly adapted purification method provides better insight into the purification HIV protease mutants which are typically challenging to express and purify. Using this newly adapted purification method, the variant protease was effectively purified from inclusion bodies and on average 50–60% of the protease were expected to be in their folded, native conformation based on the active site titration performed by ITC.

### Altered Enzyme Kinetics

The hinge region insertion variants, E355EE and L33LL, have been reported to display reduced catalytic efficiency due to the reduced turnover number of the substrate [23]. However, in this study the catalytic efficiency values for L38 were unusual considering that the catalytic efficiency (*k*_cat_/*K*_M_) was significantly increased by 1.6-fold relative to the WT protease, therefore suggesting that these insertions increase the specificity for this particular substrate. Interestingly, the addition of the four mutations (K20R, E35D, R57K and V82I) that naturally occur in L38 significantly reduces the catalytic efficiency in comparison to the WT protease. This suggests that the presence of both the insertions and mutations decrease the specificity of the protease for the substrate [28]. It is important to note that the substrate used in both studies was a WT Gag sequence and not that of the patient-derived Gage sequence. Consequently, this data suggests that while hinge region insertions may play a significant role in enzymatic properties, they need to be considered in conjunction with any other occurring polymorphisms. Drug resistance is caused by polymorphisms that alter the balance of recognition to favour binding of the substrate over inhibitors which is achieved by incorporating amino acid polymorphisms in the protease and Gag cleavage sites. The relationship between polymorphisms occurring in the protease and Gag is attributed to the fact that drug resistance mutations often result in a loss of replication capacity, but the co-evolution of the Gag substrate can restore wild type behaviour [31]. Therefore, while the affinity for inhibitors is decreased due to mutations in the protease, substrate processing is maintained by the co-evolution of the Gag substrate [32, 33]. This is demonstrated by the co-evolution of a V82A variant protease and its Gag NC/SP1 cleavage site (RQVN/FLGKIN) which binds to the protease more optimally [32].

Analysis of the Gag precursor of the L38↑N↑L clinical isolate demonstrated the co-evolution of the substrate due to the insertions and mutations present in this protease. The substrate contained mutations in the SP2/NC (T370A, M374V and R376G), NC/SP1 (E424G) and SP1/p6 (N441S) cleavage sites [28]. In addition, the L38↑N↑L Gag sequence contained a PTAPP duplication in the SP1/p6 cleavage site and multiple polymorphisms in non-cleavage sites [28]. The relationship between the dynamics of the hinge region and the substrate cleavage sites (SP1/p6 and NC/SP1) of the protease has also been established, suggesting that changes in the hinge region will be accompanied by substrate mutations [31]. The assay in these studies focused on the cleavage of the CA/SP2 site which is not mutated in the original L38↑N↑L protease. Therefore, while the cleavage of this specific site might be reduced, it is possible that the mutations in the other Gag cleavage sites may be unaffected or enhanced thereby reducing activity of the enzyme. Additionally, the K20R mutation present in the original L38↑N↑L protease increases viral fitness in mutants that has drug resistance to PIs [34] which may restore some functionality to the protease.

### Increased Protease Stability

The melting curve data for the apo proteases indicated that the two insertions were significant enough to stimulate a substantial change in the overall stability of the apo proteases. The T_m_ of the variant protease was 5 °C higher than the WT protease indicating increased stability of the apo mutated protease. This observation corresponds to literature where increased T_m_ values have been linked to multiple drug resistant mutants. An the I50L/A71V variant protease exhibited an increase of 2.2 °C in comparison to the wild type which had a T_m_ value of 61 °C [35]). The T_m_ value measured for the WT in this study corresponds to the reported value of ~ 64 °C for the subtype C protease which exhibits a higher degree of structural stability than the subtype B (~ 61 °C) protease due to the NOPs present [36]. Since the insertions occur in the hydrophobic core, the presence of an additional hydrophobic amino acid could alter the total hydrophobic surface area and side chain packing of the protease. These alterations could be responsible for the changes in the amount and strength of interactions that stabilise the native form of the protease.

**Fig. 7:**
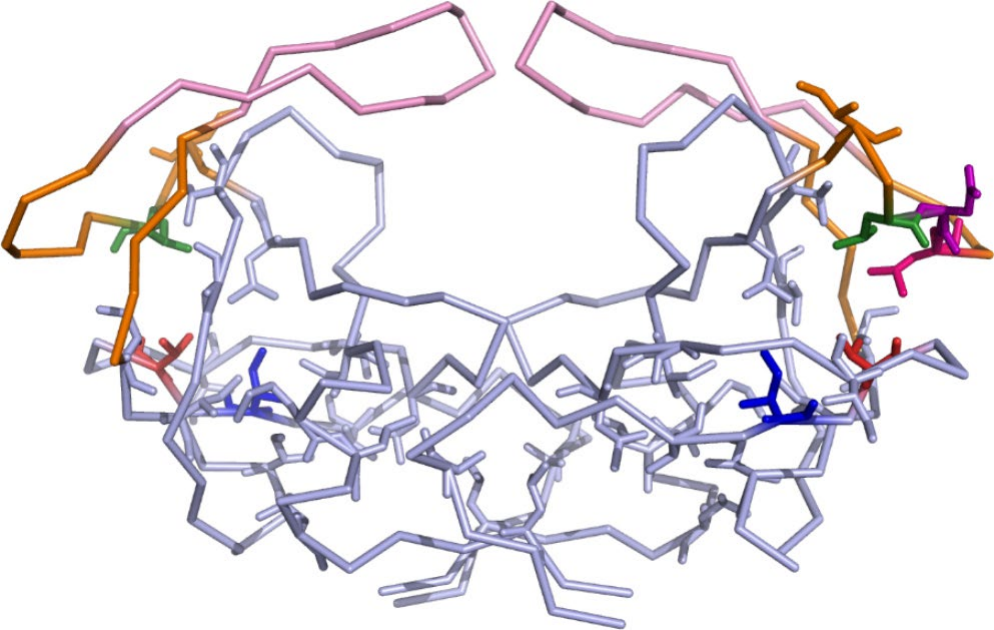
Hydrophobic amino acid side chains of the HIV-1 protease. The flaps and hinges of the HIV-1 protease is shown in pink and orange, respectively. All twenty amino acid residue side chains (L5, V11, I13, V15, I19, A22, L24, I26, L33, L38, I62, I64, I66, V75, V77, I85, M89, L90, L93, L97) involved in the hydrophobic core are indicated as sticks [7, 12]. The location of L38 is shown in green while the location of the NL double insertion is shown in pink and purple, respectively. The I13 and V15 residues of the hydrophobic core are shown in blue and red, respectively. The image was constructed using the PyMOL v0.99rc6 software using PDB ID: 3U71 as a template

Each monomer of the protease is stabilised by the hydrophobic interactions that occur within the hydrophobic core [12]. The hydrophobic sliding mechanism plays an important role in the movement of the flaps and relies on these internal hydrophobic residues to slide over one another inducing changes in flap conformers [12]. This mechanism is strictly regulated and depends principally on the internal hydrophobic surface area as well as the number of van der Waals contacts involved [13]. In the closed formation, the fulcrum residue I15 forms contacts with the cantilever residues I62, I64 and V75 and the hinge region residues M36 and L38, Fig. 7. Sliding of the hinge and cantilever regions across I15 and downwards toward the termini results in the transfer of the original contacts to I13 resulting in the opening of the flaps, Fig. 7 [12]. The L38 residue is readily involved in this mechanism, and it is possible that changes in this hinge region position may alter the stability of the protease. The increased stability caused by the insertions could be attributed to the resulting alterations within the hydrophobic core of the protease. Specifically, the addition of another leucine residue, Fig. 7, could increase the hydrophobicity of the core thereby stabilising interactions between residues involved in the hydrophobic sliding mechanism. These insertions could result in the transfer of van der Waals contacts between core residues facilitating conformational changes that stabilise the overall structure of the protease.

#### Protease Dynamics

The hinges have been identified as the key mechanistic region of the protease controlling the conformational combinations [5, 37]. Catalytic residues that are unique to certain structures are positioned close to the hinges [38]. Therefore, the hinges predominantly determine the flexibility and dynamics of the protease by triggering correlated movements of residues in the other regions [39]. These hinges display a varying degree of fluctuation (~ 10%) depending on the substrate bound [40].

The RMSD and R_g_ values indicated differences in the overall dynamic motion and degree of compactness between both proteases. The addition of the asparagine and leucine insertions resulted in an increased overall structural stability of the mutated protease. Overall, the variant was more compact and less dynamic than the WT protease. The WT displayed the highest average RMSD indicating decreased overall structural stability and, therefore, greater dynamic movements. RMSF analysis indicated that the hinge regions of the variant was slightly more flexible (3–4%) than the WT protease. The overall increase in degree of fluctuation of the variant chain B indicated that the insertions alone are sufficient to alter flexibility of the protease. Collectively, these results suggest that the insertions increase the structural stability as well as the flexibility of the protease.

### Shift to Closed flap Conformers

The flap conformations; namely, open, semi-open and closed, can be measured using either the distance between the two Ile50/Ile50’ residues or by using the distance between the Ile50 and Asp25 residues [3, 41]. The various conformations that the flaps adopt in solution allows for substrate and inhibitor binding. PIs are substrate transition-state analogues that compete for binding at the active site [42]. When an inhibitor binds, the flaps close over the active site adopting the closed conformation. With the inhibitor bound, the flaps remain in the closed conformation due to the high energetic penalty required to change the flaps to the open conformation [43]. Mutations that shift the equilibrium of these conformations may, in turn, affect drug binding. Drug resistance could be acquired by either directly changing the relative stability of the apo or inhibitor bound protease conformations to a semi-open or closed conformation, respectively [13].

Based on MD simulations, the dynamics of the flap regions of the variant protease maintained a closed conformation, whereas the WT protease sampled all three conformational states. Evidently, the insertions alone are sufficient to shift the conformation equilibrium favouring the closed conformation. The ability of the variant to stabilise and maintain a more closed conformation would decrease the accessibility of inhibitors and substrates to the active site cavity. If the flaps are mainly in the closed conformation, substrates cannot be processed efficiently. A more closed conformation would result in a decrease in substrate/protease association rates and, therefore, result in a decrease in the measured *k*_cat_ and specific activity. This correlates with our steady-state enzyme kinetics data which showed that the enzymatic properties of the variant protease is hindered.

Additionally, a decrease in RMSF for the flap regions of chain A of the variant protease was observed in comparison to the WT protease suggesting that the insertions minimise the movement of the flaps to stabilise the closed conformation of the protease. This observation correlated with our DSC data whereby the higher T_m_ values measured for the variant protease indicated an overall increase in stability. This stabilised closed conformation of the variant protease would require a substantial amount of energy to open the flaps, allow entry of inhibitors and to subsequently close the flaps to allow for binding to occur. It is suggested that this would, therefore, result in decreased drug binding of PIs due to the increased energetic penalty associated with changing the conformations of the flaps. If the closed conformation is favoured, this presents a possible mechanism by which the mutated proteases could evade drug binding.

Since the hinge and flap regions play a significant role in binding, changes in their stability and dynamics tend to affect drug binding. Except for Asp25 and Asp29, the majority of the amino acids that make up the core are hydrophobic and form hydrogen bonds with the main chain. PIs were designed to fit into the hydrophobic core and as such are mainly hydrophobic. A reduction in hydrophobic contacts is seen with drug resistant mutants [44]. As mentioned previously, the L38 residue is involved in the hydrophobic sliding mechanism and plays a role in the opening and closing of the flaps. Any polymorphisms within the hinge region could alter the hydrophobic interactions within the core and could potentially modify the accessibility of inhibitors to the active site [45].

## Conclusion

The occurrence of insertions in the hinge region of HIV protease is progressively growing and it is vital to understand the effect of these polymorphisms on the protease. In this study, we report how a double hinge region insertion of asparagine and leucine affects the enzyme kinetics, stability and dynamics of the HIV-1 protease. While the specific activity and turnover number of the variant protease were reduced in the presence of the WT Gag substrate, a future study could investigate the enzyme kinetics in the presence of the related Gag sequence. The combination of experimental and computational data correlate and indicates that these insertions stabilise the structure of the protease. The data shows that the hinge region flexibility is slightly increased and that the flaps shift to a predominantly closed conformation when the insertions are present implicating a potential mechanism for drug resistance. These findings are currently being further elucidated by characterising the effect of the insertions on drug binding.

## Electronic Supplementary Material

Below is the link to the electronic supplementary material.

Supplementary Material 1
