# Supplementary material for: HIV Protease Hinge Region Insertions at Codon 38 Affect Enzyme Kinetics, Conformational Stability and Dynamics
Source: Protein J. 2023 Jul 8;42(5):490–501. doi: 10.1007/s10930-023-10132-6 (PMC10480237; doi:10.1007/s10930-023-10132-6)
Supplement: Supplementary file 1 — Supplementary Material 1 [file 10930_2023_10132_MOESM1_ESM.docx]

**SUPPLEMENTARY FIGURE LEGENDS**


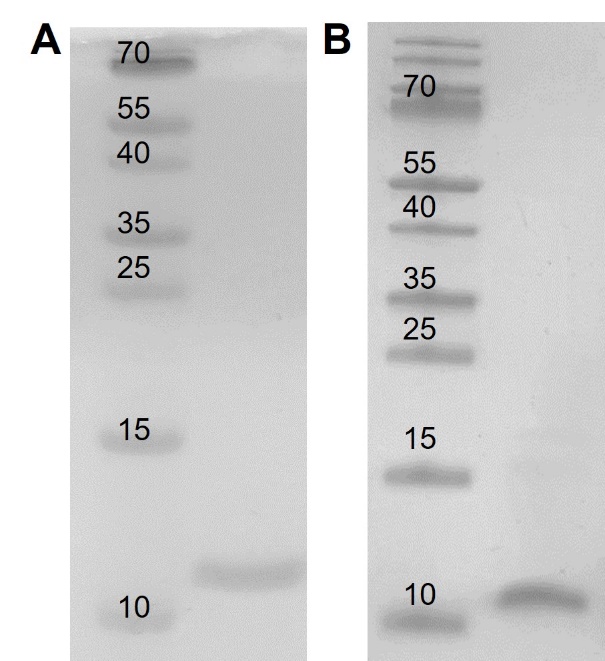


**Figure S1. Tricine 16% (w/v) SDS-PAGE of purified protease.** **A:** Polyacrylamide gel of WT protease. **B:** Polyacrylamide gel of variant protease. The molecular mass (in kDa) of each marker band is shown in the first lane of each gel.


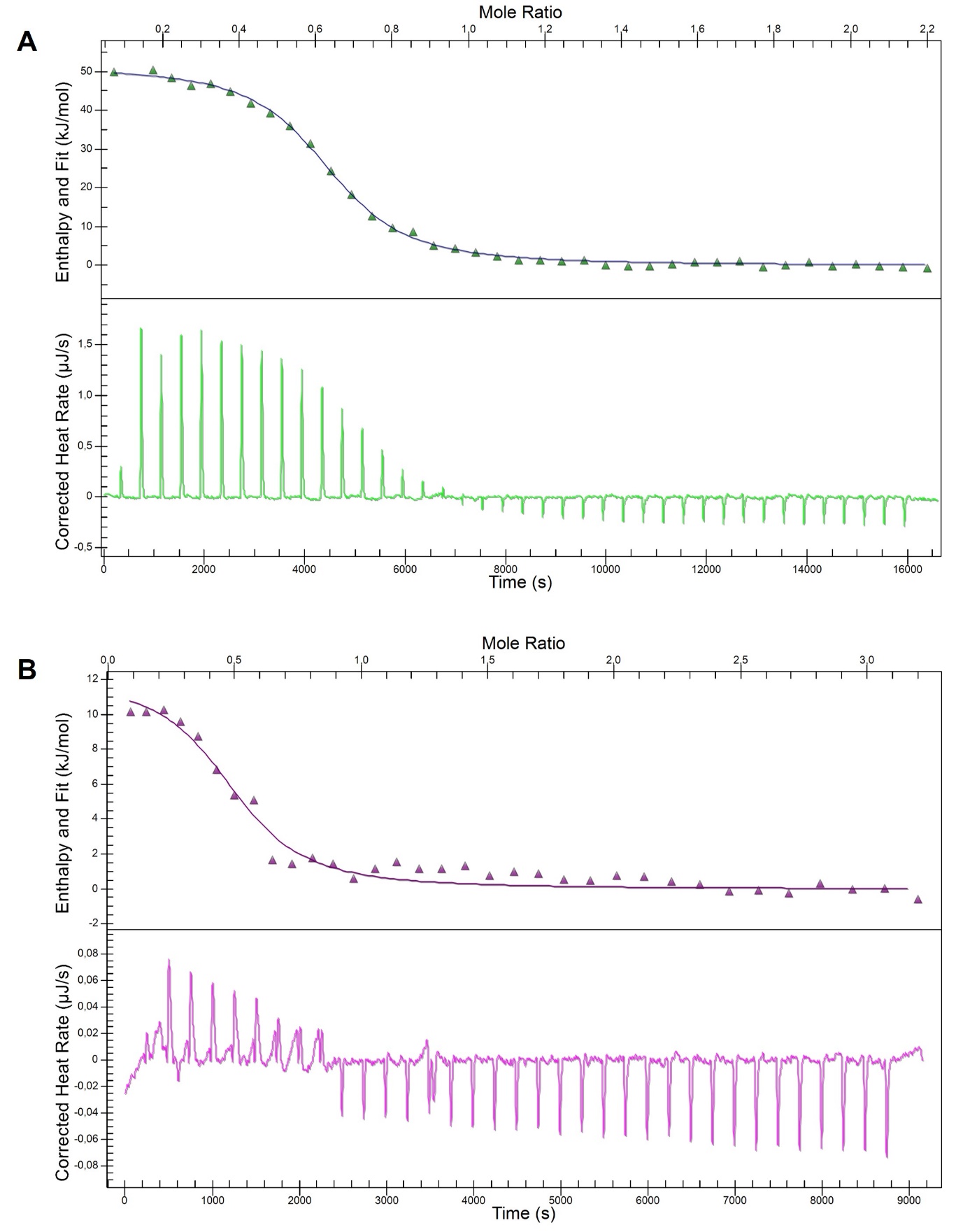


**Figure S2. Active site titrations of the WT and variant proteases**. A: Binding thermogram of the WT protease titrated with acetyl pepstatin at 20 °C. **B:** Binding thermogram of the variant protease titrated with acetyl pepstatin at 20 °C. Upper panel shows integrated heats of the peaks plotted against the molar ratio of acetyl pepstatin to C-SA protease dimer and lower panel shows baseline corrected calorimetric data.


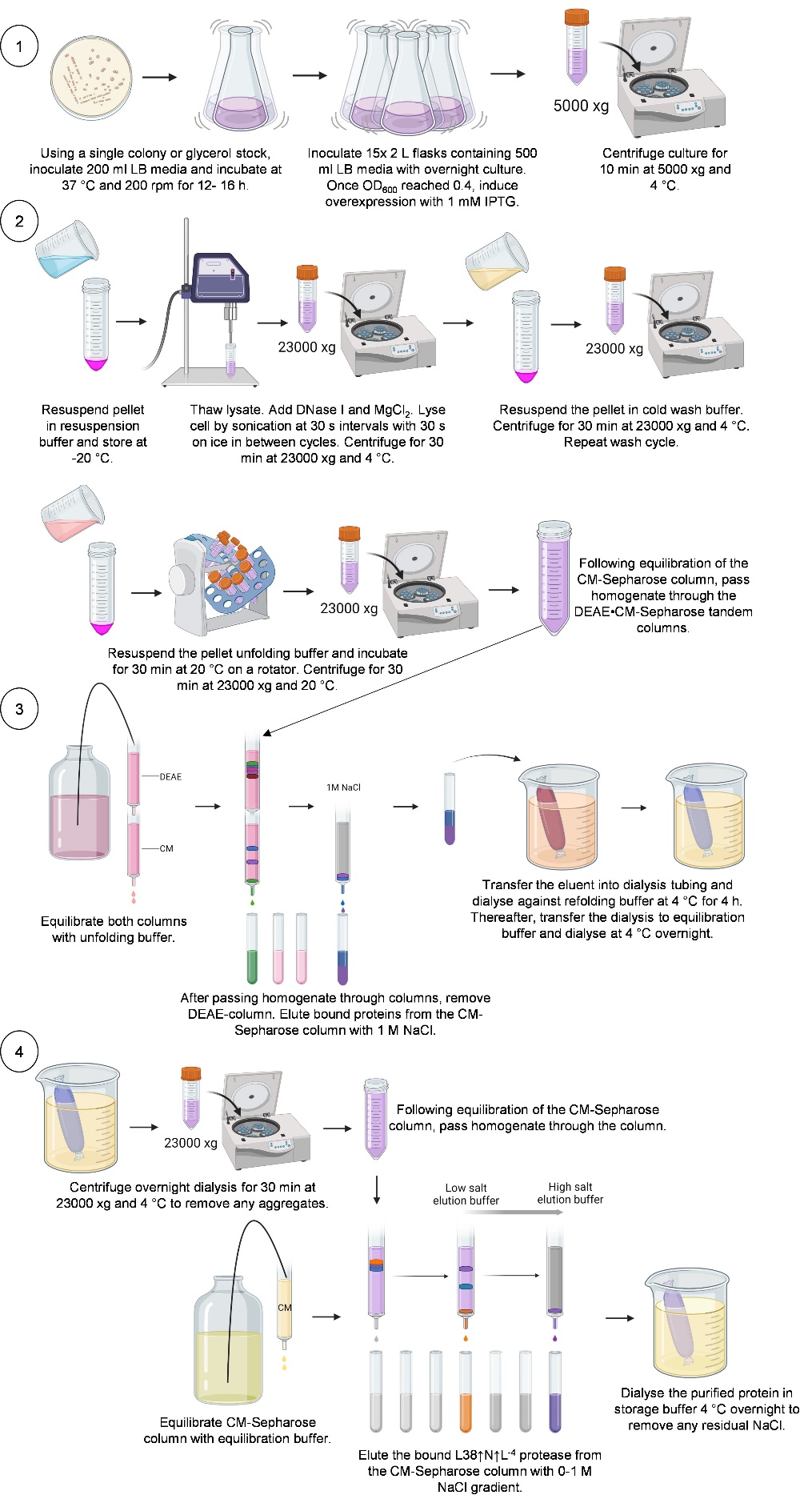


**Figure S3.** **Overexpression and purification of the variant** **protease.** Step 1 illustrates the expression of the protease. Steps 2 illustrates the lysis, washing and unfolding of the lysate. Step 3 illustrates the first purification with both anion and cation exchange chromatography. Step 4 illustrates the second purification with cation exchange chromatography. Image created with BioRender.com.
